# On the triad of air PM pollution, pathogenic bioaerosols, and lower respiratory infection

**DOI:** 10.1007/s10653-021-01025-7

**Published:** 2021-07-08

**Authors:** Tangtian He, Ling Jin, Xiangdong Li

**Affiliations:** 1 Department of Civil and Environmental Engineering, The Hong Kong Polytechnic University, Hung Hom, Kowloon, Hong Kong; 2 The Hong Kong Polytechnic University Shenzhen Research Institute, Shenzhen, 518057 China

**Keywords:** Lower respiratory infection, Infectious pathogens, Air pollution, Biogeochemical factors, Spatiotemporal patterns

## Abstract

Airborne particulate matter (PM) pollution, as a leading environmental health risk, causes millions of premature deaths globally every year. Lower respiratory infection (LRI) is a sensitive response to short-term exposure to outdoor PM pollution. The airborne transmission of etiological agents of LRI, as an important pathway for infection and morbidity, bridges the public health issues of air quality and pathogen infectivity, virulence, resistance, and others. Enormous efforts are underway to identify common pathogens and substances that are etiological agents for LRI and to understand the underlying toxicological and clinical basis of health effects by identifying mechanistic pathways. Seasonal variations and geographical disparities in the survival and infectivity of LRI pathogens are unsolved mysteries. Weather conditions in geographical areas may have a key effect, but also potentially connect LRI with short-term increases in ambient air PM pollution. Statistical associations show that short-term elevations in fine and coarse PM lead to increases in respiratory infections, but the causative agents could be chemical or microbiological and be present individually or in mixtures, and the interactions between chemical and microbiological agents remain undefined. Further investigations on high-resolution monitoring of airborne pathogens in relation to PM pollution for an integrated exposure–response assessment and mechanistic study are warranted. Improving our understanding of the spatiotemporal features of pathogenic bioaerosols and air pollutants and translating scientific evidence into effective policies is vital to reducing the health risks and devastating death toll from PM pollution.

**Graphical abstract:**

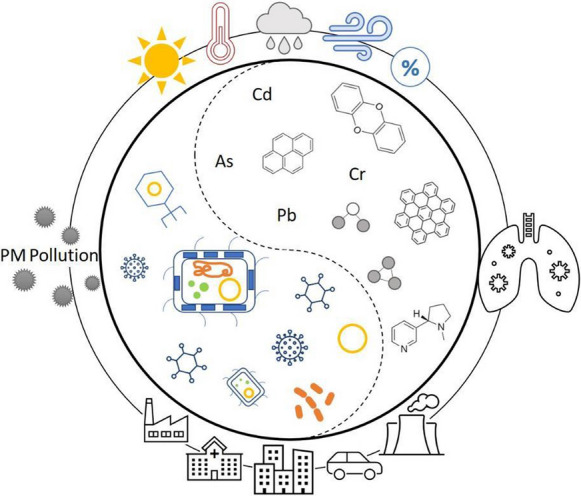

## Introduction

Global exposure to airborne particulate matter (PM) pollution has been increasing by 20% over the last two decades. More than 90% of the world’s population now live in areas that exceed the WHO’s Air Quality Guideline. According to recent estimates by the WHO, air PM pollution, as a leading environmental health risk, causes 4.2 million premature deaths globally every year (http://www.who.int/airpollution/en/).

Among the major morbidities/mortalities attributable to outdoor air pollution, upper and lower respiratory infections are sensitive responses to short-term exposure to outdoor air pollution by respirable PM, including coarse (PM_2.5–10_; aerodynamic diameter of between 2.5 and 10 µm) and fine particulate matter (PM_2.5_; aerodynamic diameter of less than 2.5 µm) (Burnett et al., 2018; Horne et al., 2018). From a toxicological perspective, the mechanistic nature of such statistical associations is vague, particularly in terms of the causative agents, whether be chemical or microbiological, individually or in mixtures, that increase the infection risks for mass populations.

**Fig. 1:**
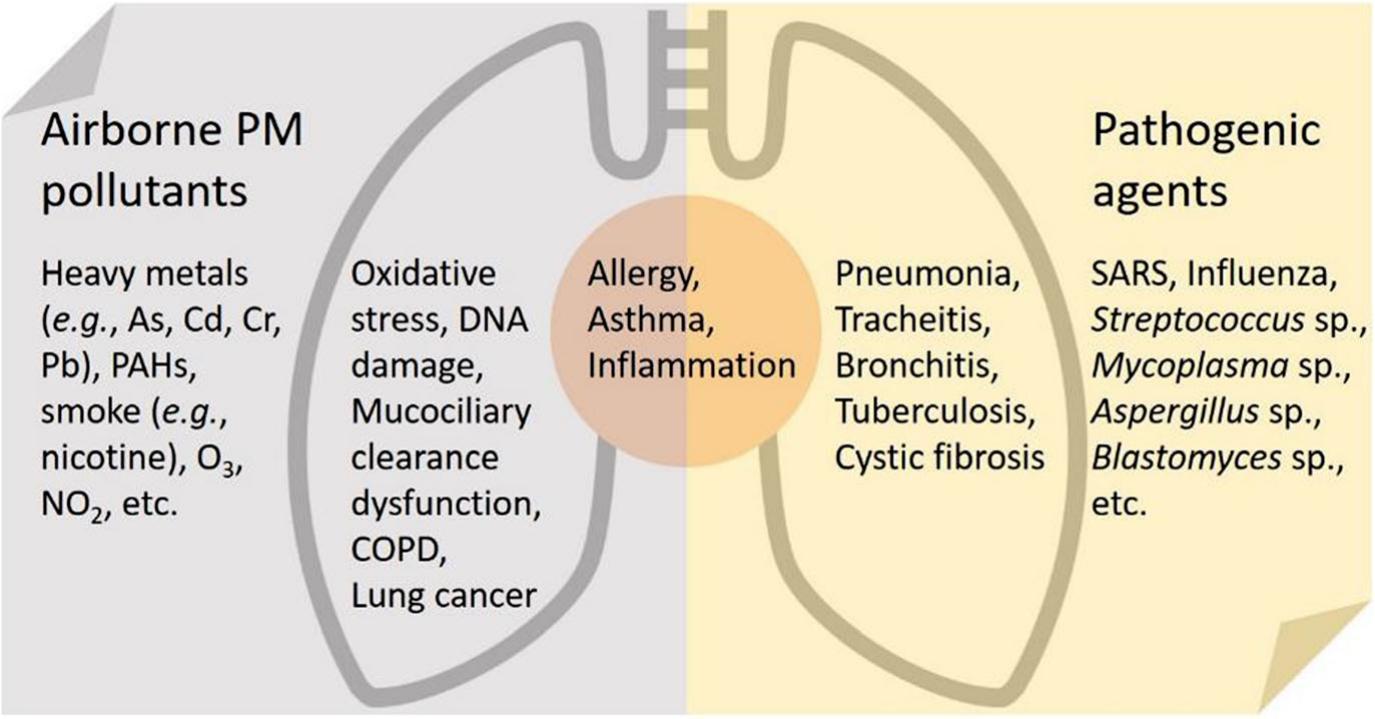
Potential interactions of air pollution and pathogenic bioaerosols for LRI (Aquilina et al., 2021; Armstrong et al., 2004; Cao et al., 2020; Conticini et al., 2020; Fernstrom & Goldblatt, 2013; Galli et al., 2008; Kim et al., 2015; Lee et al., 2012, 2015; Liang et al., 2020; Moorthy et al., 2015; Pavia, 2011; Simoes et al., 2000,2006; Stejskal, 2014; Tchounwou et al., 2012; Wu et al., 2020; Zhong et al., ; Zhu et al., 2020; Zuo et al., 2020). COPD, chronic obstructive pulmonary disease; PAHs, polycyclic aromatic hydrocarbons; SARS, severe acute respiratory syndrome

The clinical understanding of the etiological agents offers insights into this issue. Lower respiratory infection (LRI) includes infections of the lungs and alveoli (pneumonia) and the airways (bronchitis and bronchiolitis) (Fig. 1), which are a leading cause of morbidity and mortality in children and adults worldwide. LRI often has characteristic seasonal peaks. The seasonal features suggest that weather may have a key effect, but also potentially connecting LRI with short-term increases in ambient air PM pollution (Fernstrom & Goldblatt, 2013; Moriyama et al., 2020). Whether ambient PM serves as a transmission vector for the etiological infectious agents is important to the fundamental understanding and effective control of LRI and other health outcomes in many parts of the world.

Despite the scattered understanding of air pollution, pathogenic bioaerosols, and lower respiratory infection in different disciplines, critical barriers are yet to be broken at the biogeochemistry–health interface (Burnett et al., 2018; Fernstrom & Goldblatt, 2013; Horne et al., 2018; Jin et al., 2017; Moriyama et al., 2020):

1. By what mechanisms do the causative agents of LRI work to pose a risk to the public?
2. How do seasonal/weather influences shape the spatiotemporal features of LRI?
3. Do the connection and interplay between chemical and microbiological agents in PMs lead to an increase in LRI?

To the benefit of public health in consideration of socioeconomic burdens, proactive countermeasures against LRI at the environmental origins are becoming increasingly important than reactive diagnosis and treatment at the clinical end. Concerted efforts and dialogues are thus required among experts from a wide range of disciplines, such as immunology, epidemiology, microbiology, toxicology, and (bio)geochemistry. With the above critical barriers in mind, we aim to provide holistic perspectives on the research needs to disentangle the interplay between airborne PM pollution, pathogenic bioaerosols, and LRI.

## Current advances

### Causative agents as an integral part of PM

LRI refers to any infections in the lower respiratory tract, including pneumonia, bronchitis, and tuberculosis. The etiological agents for LRI transmitted via airborne particles and droplets have largely been established in medical and clinical microbiology (Fig. 1). The major syndromes include infection of the lungs and alveoli (pneumonia) and of the airways (bronchitis and bronchiolitis) (Pavia, 2011). They are a leading cause of morbidity and mortality in children and adults worldwide. Viruses responsible for LRI include respiratory syncytial virus, influenza, parainfluenza, adenovirus, severe acute respiratory syndrome (SARS) coronavirus, and human metapneumovirus (Fernstrom & Goldblatt, 2013; Pavia, 2011; Zuo et al., 2020). Bronchiolitis is inflammation or swelling of the small airways in the lungs. It is the most common LRI in children, with 50–90% of cases caused by respiratory syncytial virus. It occurs primarily in children younger than two years old and most commonly in babies between 3 and 6 months old. It is also the most common cause of hospitalization in the first two years of life. Coronavirus-related infectious diseases emerged from the beginning of the twenty-first century. The highly lethal SARS-CoV-1 caused an outbreak of SARS between 2002 and 2004, with a global total of 8,098 reported cases and 774 deaths and a case fatality rate of 9.7%. In comparison, the deadly Middle East respiratory syndrome coronavirus (MERS-CoV) emerged in 2012 and is currently not presenting a pandemic threat. It has caused 2,494 reported cases and 858 deaths in 27 countries and has a very high case fatality rate of 34%. The novel coronavirus SARS-CoV-2 is less deadly but far more transmissible than MERS-CoV or SARS-CoV. Emerging in 2019, SARS-CoV-2 is causing an ongoing global pandemic of what we now know as COVID-19, with 181,930,736 confirmed cases, 3,945,832 deaths and a case fatality rate of 2.2% (as of 1 July 2021). While viruses account for most cases of respiratory tract infection, bacteria and fungi are also often implicated in more severe episodes of infection (Fernstrom & Goldblatt, 2013; Simoes et al., 2006). The commonest cause of community-acquired pneumonia is *Streptococcus pneumoniae*. Atypical pneumoniae are caused by such agents as *Haemophilus influenzae*, *Legionella pneumophila*, *Moraxella catarrhalis*, *Mycobacterium tuberculosis*, *Mycoplasma pneumoniae,* etc. Fungi, such as *Aspergillus* sp. and *Blastomyces* sp., are less common causes but important in immunocompromised people. Out of concern for critical public health issues, studies on the bacterial and fungal agents of LRI are being devoted not only to determining causative pathogen phenotypes, but are also focusing on the important aspects of virulence and resistance. For instance, studies have recently demonstrated that ambient PM_2.5_ serves as a pathway for the transmission of antibiotic resistance genes (He et al., 2021; Xie et al., 2019), and some geographically independent common taxa members across regions that could be potential host bacteria of antibiotic resistance genes have been identified. These genes can be horizontally transferred from one bacterial species to another via three canonical mechanisms, namely conjugation, transduction, and transformation, which map to antibiotic resistance genes in bacteria, phages, and free DNA, respectively (von Wintersdorff et al., 2016). The three fractions of antibiotic resistance genes have been recently discovered in airborne PM_2.5_, indicating multiply gene transfer pathways from ambient air to human airways (He et al., 2021).

The physiochemical compositions of airborne PM and the related health impacts have been widely studied (Jin et al., 2017; Li et al.,^ 2019^; Kelly & Fussell, 2015). Typical toxic chemical components in airborne PM, including heavy metals (e.g., As, Cd, Cr, and Pb) (Kim et al., 2015; Tchounwou et al., 2012), polycyclic aromatic hydrocarbons (PAHs) (Armstrong et al., 2004; Moorthy et al., 2015), and cigarette smoke-derived nicotines (Aquilina et al. 2021; Zhong et al. 2000) 2004,2015, are known to induce biological perturbations (e.g., oxidative stress, DNA damage, inflammation) that are essential for the development of certain respiratory diseases (Fig. 1). Region-specific insights have been introduced into the quantitative contribution of major source categories to the mixture effects or combined health risks of typical PM_2.5_-associated pollutants (Jin et al., 2019; Xie et al., 2020). A series of in vitro and in vivo toxicological studies have identified reactive oxygen species and pro-inflammatory cytokines as sensitive biochemical markers responding to air PM exposure (Lee et al., 2015). Through proteomic profiling of mouse lungs, the biological pathways have been further explored, including the involvement of WNT/*β*-catenin signaling pathways in the toxicity of PM_2.5_. The suppression of *β*-catenin levels, the activation of caspase-3, alveolar destruction, as well as the production of IL-6, TNF-*α*, and IFN-*γ*, have been observed in the lungs in vitro and in vivo in response to PM_2.5_. Recent research has found that endotoxin, a potent immune-stimulatory component in the outer membrane of gram-negative bacteria, contributes a large proportion of the in vitro toxicity of urban PM_2.5_ from China to human lung cells, beyond the well-known chemical toxicants, such as heavy metals and PAHs (Jin et al., 2019). Complete dissection of component- and source-specific contribution to PM-induced pulmonary toxicities would advance the further understanding of the chemical–microbial interplay on the LRI disease development (Fig. 1) (Cao et al., 2020; Galli et al., 2008; Lee et al., 2012; Stejskal, 2014).

From a biogeochemical perspective, bioaerosols, including bacteria, fungi, viruses, and cell debris, are a subset of atmospheric particles. Together with chemical aerosols (e.g., inorganic and organic components), they form complex mixtures of PM and have a profound impact on human health. In contrast to extensive physicochemical characterizations, the understanding of the microbiological dimension of such inhalable cocktails is still in its infancy (Jin et al., 2017). Materials of biological origin, e.g., pollen, bacteria, fungi, and viruses, are estimated to contribute as much as 25% to the atmospheric aerosol (Jaenicke, 2005), which may be responsible for various diseases and allergies. The aerosolization of soil–dust and spray facilitates the long-range transport of bacteria and viruses across the atmosphere (Reche et al., 2018). A notable example is the detection of higher loads of ambient influenza and avian influenza viruses during Asian dust storms compared to background days, suggesting the long-range transport of viruses by dust storms (Chen et al., 2010). A prominent example is the discovery and categorization of microbial community structures, including two of the major LRI pathogens (i.e., *Streptococcus pneumoniae* and human *adenovirus C*) and other microbial allergens and pathogens in respirable ambient PM during the well-known winter haze events in Beijing (Cao et al., 2014). Interestingly, these pathogens appeared to increase in relative abundance as levels of PM loadings increased by an unresolved mechanism. The high relative humidity during the periods of haze may have contributed to particle growth and aggregation through water uptake and the promotion of aqueous redox chemistry (e.g., the oxidation of sulfur dioxide to sulfate) (Cao et al., 2014). This also suggests that most of the particles had a high-water content during polluted days, which might favor the survival of microbes (Stanier et al., 2004). However, the viability or infectivity of these detected pathogen species, like all other members of the microbial community, has not been well understood.

### Aerosolization: critical for agent transmission

Although LRI pathogens have been studied comprehensively when they result in illnesses requiring hospitalization, their environmental properties with implications for exposure at the air–human interface have not been well investigated. LRI pathogens may be transmitted among humans in three ways: (1) by direct contact with infected individuals; (2) by contact with contaminated objects (called fomites, such as toys and doorknobs); and (3) by inhalation of pathogen-laden droplets of various sizes. The contribution of each mode to the overall transmission of pathogens is not clearly known. Respiratory transmission depends upon the origin of droplets and airborne infections. Speaking and normal breathing all produce droplets of airborne microorganisms, while coughing and sneezing lead to the more forceful expulsion of greater amounts of infectious particles. While coughing may produce several hundred particles, a sneeze can generate up to 40,000 droplets of respirable size, ranging between 0.5 and 12 μm in diameter (Fernstrom & Goldblatt, 2013). Particles produced by these activities are of different sizes. The largest droplets fall to the ground within a few meters and can transmit infection only to those in the immediate vicinity. Other droplets can travel a distance dependent on their size. Those droplets of 1–4 µm in diameter are called “droplet nuclei” or aerosols; these remain suspended in the air for very long periods and may not only travel long distances, but also reach the lower respiratory tract (Fernstrom & Goldblatt, 2013). Chamber and modeling studies have shown that aerosol transmission could account for approximately half of all transmission events, for example, of influenza viruses (Milton et al., 2013). While aerosols can travel long distances and stay in the air for long periods, it is likely that most aerosol-mediated transmissions occur at short ranges and soon after exhalation, because these particles are easily dispersed and diluted.

In the global fight against the COVID-19 pandemic, researchers in the engineering, physical, and chemical sciences have contributed to research on the aerosol dissemination of SARS-CoV-2, which has provided mounting evidence to suggest that virus-laden aerosols causing SARS-CoV-2 lung infections play an important role in the rapid spread of the disease (Zuo et al., 2020). Through modeling and simulation studies, e.g., computational fluid dynamics (CFD) and molecular dynamics simulations, key steps in the airborne transmission pathway have been hypothesized and suggested: starting from the initial aerosol ejection by a cough or exhalation, to the deposition of the virus-laden aerosols in the lung, and to the binding of the S protein of SARS-CoV-2 to the ACE2 receptor on the surface of goblet cells in the lung epithelium. These involve the exchange of mass, energy, and momentum among virus, aerosol particles, and the ambient environment. Apart from a lack of direct clinical evidence, the remaining challenge is to identify relevant contributing physicochemical processes and resolve them under the framework of well-rounded computational models. Following current research strengths on SARS-CoV-2, the expectation is that a more comprehensive understanding of the complete pathway for the airborne transmission of etiological LRI agents will be achieved.

### Spatiotemporal features of airborne agents

During the droplet/aerosol and contact dissemination period, the survival and infectivity of pathogens are certainly affected and governed by various types of environmental conditions, e.g., temperature and humidity (Fernstrom & Goldblatt, 2013; Moriyama et al., 2020; Zuo et al., 2020). Evidence of these effects can be seen from the fact that infectious respiratory diseases and environmental factors usually exhibit strong seasonal cycles and geographical differences. However, these environmental factors influence viral, bacterial, and fungal particles in different ways.

Viruses are incapable of autonomous reproduction. Thus, the temporal pattern of viruses is subjected to more complicated conditions, such as seasonality in the survival of pathogens outside their hosts, the behavior of the host, and the immune function of the host (Kormuth et al., 2018, 2019; Moriyama et al., 2020). Taking influenza as a representative of viruses, influenza epidemics typically peak during the winter season in temperate regions and during the rainy season in tropical regions, in association with cool and dry conditions or very humid conditions (Moriyama et al., 2020). In studies involving kinetic modeling, animal models (e.g., ferrets), and chamber examination, solid evidence has been found of the importance of temperature and relative humidity in the transmission of influenza (Kormuth et al., 2018, 2019; Zhou et al., 2018), and of the ability of influenza viruses to remain infectious for extended periods of time in aerosols and droplets across a wide range of levels of relative humidity. The viability of these viruses in droplets was found to have increased both at higher (> 60%) and lower (< 40%) levels of relative humidity (Moriyama et al., 2020), but not all viruses would respond identically to relative humidity in the atmosphere. It has been reported that highly transmissible seasonal influenza viruses are less sensitive to decay under midrange levels of relative humidity in droplets and that the presence of host airway surface liquid can protect influenza viruses from relative humidity-dependent decay in suspended aerosols and stationary droplets (Kormuth et al., 2018, 2019). Similar to influenza viruses, current research studies suggest that SARS-CoV-1 and 2 in the droplets can survive for 3 h in the air (van Doremalen et al., 2020), while low temperatures and an ideal air humidity range could further increase their lifespan, e.g., both high and low levels of relative humidity are favorable to the transmission of SARS-CoV-2 (Moriyama et al., 2020; Zuo et al., 2020).

In comparison with viruses, bacteria can survive a wider range of temperatures and humidity levels. Temperatures above 24 °C are required to reduce the survival of airborne bacteria, with a number of gram-negative and gram-positive bacterial pathogens showing evidence of the relationship (Fernstrom & Goldblatt, 2013). However, determining the rates of survival of airborne bacteria appears to be a more complicated process than determining those of viruses. For instance, the survival of aerosolized gram-negative bacteria (including *Pseudomonas* sp., *Enterobacter* sp., and *Klebsiella* sp.) has been reported to be greatest at high levels of relative humidity, but airborne gram-negative bacteria (e.g., *E. coli* and *Salmonella* sp.) are reported to not survive well at increased levels of relative humidity, while some airborne gram-positive bacteria (*Staphylococcus albus* and *Streptococcus pneumoniae* (type 1)) survive poorly at intermediate levels of relative humidity. Seasonal variations in airborne fungal and spore concentrations associated with common environmental settings, including ambient temperature, relative humidity, precipitation, and wind speed, have also been suggested in many studies (Fernstrom & Goldblatt, 2013). In general, fungi and their spores appear to be more resilient than bacteria and viruses, being able to endure greater stresses due to de- and re-hydration, as well as UV radiation. The effects of atmospheric physiochemical conditions on airborne bacteria structure and resistance are also of research interest. For instance, antibiotic resistance profiles and dominant DNA fractions of antibiotic resistance genes in airborne PM_2.5_ show geographical disparities (He et al., 2021; Xie et al., 2019). In cities like Hong Kong, antibiotic resistance genes in airborne PM_2.5_ are predominantly carried by phages, while in Beijing and Hangzhou, antibiotic resistance genes are more abundant in free and bacterially associated fractions, respectively. Meteorological factors and trace gases, including temperature, UV, and ozone, play important roles in shaping spatiotemporal disparities in the airborne dissemination of antibiotic resistance genes. As free DNA and phages (quasi-ultrafine particle, less than or equal to 0.1 µm) are distinguished from bacteria (between 1 and 10 µm) in size distribution, the geographically disparate abundance of antibiotic resistance genes in these fractions would result in geographical differences in inhalation and deposition in human lungs (He et al., 2021).

Apart from atmospheric physiochemical conditions, the local and long-range dispersion of sources also contribute greatly to shaping spatiotemporal disparities in airborne LRI agents. A recent study using the concept of bioanalytical equivalent (BEQ) and mixture-toxicity experiments and modeling also found that transition metals (dominated by Fe, Cu, and Mn) and PAHs (dominated by dibenzo[a,l]pyrene) accounted for up to 40% of the oxidative stress in the human lung cells exposed to PM_2.5_ collected from Beijing and Guangzhou (Jin et al., 2019). The differential mixtures of transition metals and PAHs partially explained the disparities in toxicity potency at equal concentrations of PM_2.5_ between the two cities. To trace the local, regional, and remote sources of pollutants, integrated chemical approaches using stable isotopes, molecular tracers, an air backward trajectory analysis, dynamic transport models, and receptor models can be employed. The long-range transport and vertical convection of air PM and selected particle-bound chemicals have been demonstrated in regional studies (Luo et al., 2014; Ming et al., 2017; Xu et al., 2012).

In addition to PM chemical–microbial compositions and toxicity, combined field and laboratory studies of the transmission of respiratory viruses in the community to resolve influenza transmission mechanisms, including at the animal–human interface, have been designed and implemented. For example, a transmission chamber that separates virus-laden particles in the air by size has been established to study airborne particles that mediate influenza transmission in ferrets (Zhou et al., 2018). The results provided direct experimental evidence of influenza transmission via droplets and fine droplet nuclei, albeit at different efficiencies. This transmission device can also be applied to elucidate the mode of transmission of other respiratory pathogens. Contrary to the prevailing paradigm that humidity modulates the stability of respiratory viruses in aerosols, a recent study found that viruses supplemented with material from the apical surface of differentiated primary human airway epithelial cells remained equally infectious for 1 h at all of the levels of relative humidity that were tested (Kormuth et al., 2018, 2019). The sustained infectivity was observed in both fine aerosols and stationary droplets. These results have significant implications for understanding the mechanisms of the transmission of influenza and its seasonality in the environment.

Overall, the current findings suggest that atmospheric physiochemical factors and source emissions play an important role in the air transmission of pathogens by affecting the properties, e.g., viability and resistance, as well as the differentiation of PM_2.5_ composition profiles, which further cause spatiotemporal disparities among airborne LRI agents. However, most of the current evidence was obtained based on field observations and statistical correlations. More realistic approaches are warranted to study the mechanistic nature of the pathways of transmission.

### Pathogenic bioaerosols and air pollution duet

The sized particle fractions for LRI indicate not only the potential sources of emission (Cao et al., 2013), but also the specific regions of the human respiratory tract where respirable particles can be deposited at quantitatively predictable efficiencies (Fernstrom & Goldblatt, 2013). This is of particular relevance to the onset of LRI, which affects the respiratory system below the throat. Short-term elevations of fine and coarse particulate matter have been leading to increases in LRI around the world (Leecaster et al., 2011; Passos et al., 2014; Qiu et al., 2014). The mechanistic nature of such statistical associations is, however, unclear from the toxicological perspective, particularly in terms of the true causative agents that underlie the development of LRI among large urban populations. Such causative agents could be chemical or microbiological and be present individually or in mixtures.

Evidence that the inhalation of ambient air pollutants increases a population’s vulnerability to airway infection dates back to at least the Great London Smog (Bell & Davis, 2001). During that period, deaths from pneumonia, a major syndrome of LRI, increased threefold. Notifications for pneumonia also increased 1.4-fold during the smog event itself and 2.4- to 2.7-fold in the subsequent two weeks compared with the corresponding weekly average during the period of 1947–1951. Similar to the pattern of pneumonia diagnoses during and after the 1952 London smog event, Horne et al. recently found that the risk for children younger than two years of developing LRI increased during the week following elevated PM_2.5_ and that this increased risk persisted for three weeks after the event (Horne et al., 2018). An association between exposure and increased LRI risk was also found in the subgroup of young children with proven respiratory syncytial virus infection.

Taking the global pandemic of SARS-CoV-2 as an example, a case fatality rate of over 15% for COVID-19 patients was reported in early 2020 in countries like Italy, and a considerable number of epidemiological studies pointed to interactions between the coronavirus epidemic and air pollutants, e.g., PM_2.5_, O_3_, and NO_2_, as having potentially contributed to the devastating toll (Fig. 1) (Conticini et al., 2020; Liang et al., 2020; Zhu et al., 2020). For instance, a regression analysis showed that an increase of 1 μg/m^3^ in PM_2.5_ is associated with an 8% increase in the COVID-19 death rate (Wu et al., 2020). Air pollution can impair the first defensive line of the upper airways, damage ciliary structure and function to impact mucociliary clearance, and lead to lower respiratory illness (Fig. 1) (Cao et al., 2020); hence, living in an area with high levels of pollutants may trigger the development of chronic respiratory conditions that are favorable to the development of acute LRI. Moreover, long-term exposure to air pollution leads to chronic inflammatory stimulus, even in young and healthy subjects. However, the above evidence was obtained in modeling investigations. Experimental and epidemiological studies are urgently needed to evaluate the role of atmospheric pollution in cases of illness and death in epidemics.

Despite the improvement in air quality from the days of the notorious smog events in many industrialized towns and cities (e.g., Great Smog of London, Los Angeles photochemical smog) to the current situation (e.g., medium to low PM pollution levels in many parts of the world, including Hong Kong), the association between LRI and PM appears to persist. Globally, the elevated hazard ratio of LRI in response to PM_2.5_ exposure can still occur at lower levels, those that are close to the WHO’s air quality guidelines (Burnett et al., 2018). Although mechanistic research efforts have been directed at identifying the chemical mixtures and biological mechanisms responsible for potential infection through PM exposure (Harder et al., 2001), there is arguably an alternative hypothesis, namely that the pathogens causing LRI are themselves part of the respirable ambient PM in proportionally increasing abundance, particularly during pollution episodes that coincide with an elevated risk to sensitive urban populations of developing LRI. Connections between pathogenic bioaerosols and air pollutants have the potential to aggravate respiratory infections; thus, their co-effects on human health warrant further investigation.

### The way forward

**Fig. 2:**
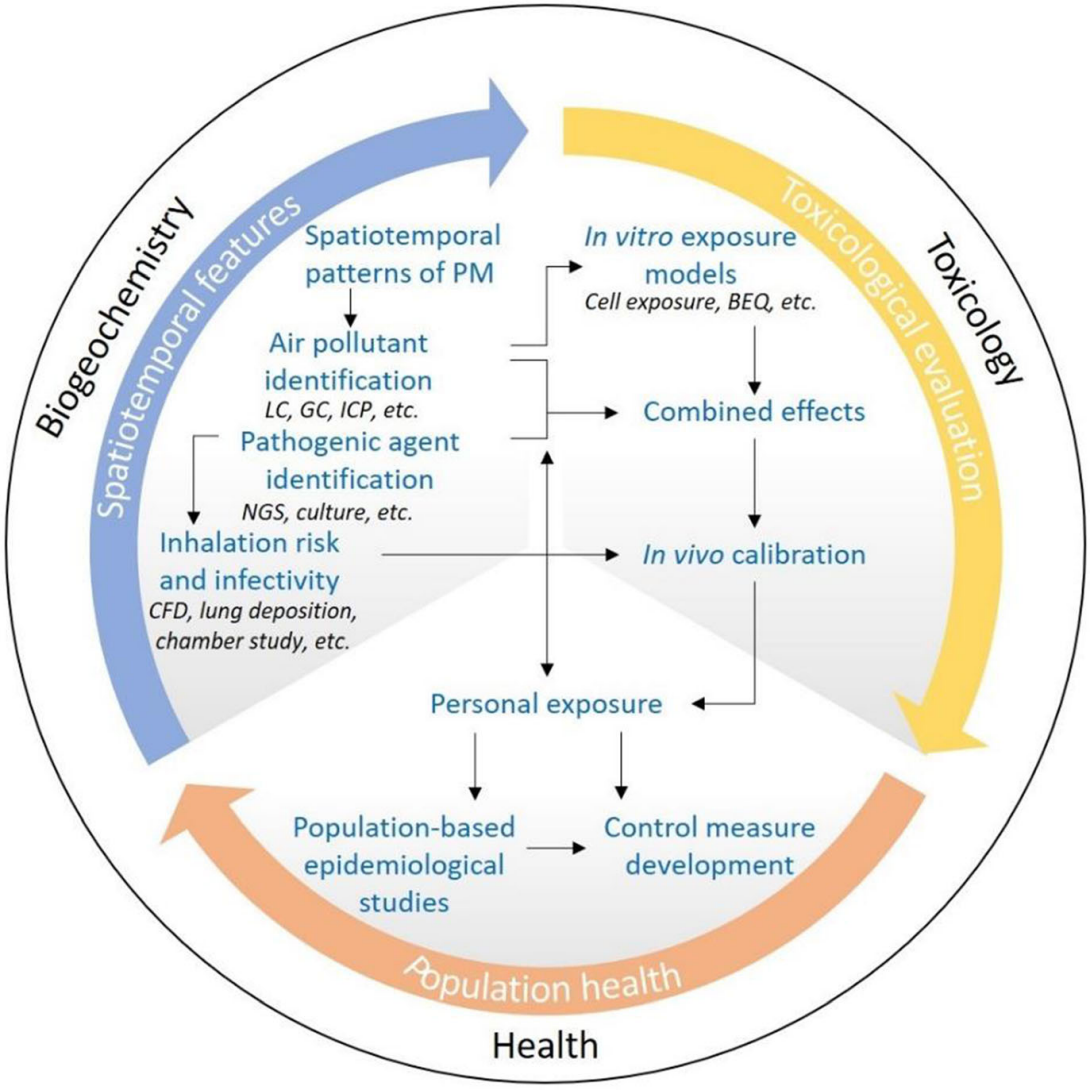
Proposed methodological framework for holistic assessments of the effects of pathogenic bioaerosols and air pollution on LRI and human health from a molecular basis to the epidemiological consequences. LC, liquid chromatography; GC, gas chromatography; ICP, inductively coupled plasma; NGS, next-generation sequencing; CFD, computational fluid dynamics; BEQ, bioanalytical equivalent

The seasonal dynamics of airborne pathogens associated with geographical-specific PM profiles taking climatic features and pollution gradients into consideration, remain largely unknown. This knowledge is a pre-requisite for the mapping of exposures to airborne pathogens in relation to air PM pollution for an integrated exposure–response and public health assessment. Understanding such mechanisms requires a seamless connection between the source–pathway–receptor continuum at the environmental end and the hazard–exposure–disease continuum at the health end (Mahoney et al., 2015; Stewart & Wilkinson, 2020). For example, it is imperative to answer whether infectious pathogens are present in high enough concentrations in ambient PM to contribute sufficiently to the dose that is causing an epidemic of LRI in places with large populations. Further questions should follow, such as whether exposure airborne PM and associated toxic constituents may impair the host innate immunity and render certain population vulnerable to pathogen infection. Anchoring these overarching questions are the unresolved environmental mechanisms underlying seasonal and geographical disparities in dissemination sources, exposure regimes, and hence health outcomes of air pollution and airborne pathogens (Fig. 2). This is where environmental (bio) geochemists can particularly contribute their expertise to resolve the environmental pillar of the host–agent–environment triad (Cohen & Powderly, 2004). Such a framework encourages and requires multidisciplinary synergies to unravel the chemo- and bio-diversity of what we breathe across spatiotemporal scales, exposure sequences of multiple hazards, and combined toxic effects that result in disease progression over time.

Recent decades have seen the development of state-of-the-art technologies in biogeochemistry and bioinformatics. These robust analytical tools, e.g., next-generation sequencing (a high-throughput technology that enables sequencing of millions of small fragments of DNA in parallel) (Behjati & Tarpey, 2013; Quince et al., 2017) and novel culture techniques (Lewis et al., 2020), are favorable for the investigation of the common and emerging chemical and microbiological agents of respiratory infection in respirable particles, and the assessment of the infectivity of the detected viral pathogens and the viability and antimicrobial resistance of the detected bacterial pathogens. Apart from modeling and simulations, laboratory-based chamber studies, in situ monitoring, and in vivo exposure models are also realistic approaches to investigating the influence of environmental conditions (e.g., temperature, relative humidity, PM, and O_3_) on the abundance of active pathogens and the combined effects of the chemical and microbiological agents.

The prior research spans the environmental, epidemiological, and medical aspects of the infection–pathogen–pollution link, which has prompted us to cross research fields in search of answers to the more crystallized scientific question: whether ambient respirable particles in certain environmental settings at peak seasons contain high enough concentrations of active LRI pathogens, and hence enhance the mechanistic link between LRI encounters and air PM pollution? Current research strengths call for an interdisciplinary research work to achieve a more comprehensive understanding of the environmental aspects of the LRI causative pathogens that connect the ambient air environment and human exposure events prior to the development of disease. Therefore, the environmental aspects of respiratory pathogens should be integrated into the complex link between respiratory infectious diseases and ambient PM pollution, so that the appropriate control measures can be formulated to best ensure the health of the public.
